# The impact of multiple non-pharmaceutical interventions for China-bound travel on domestic COVID-19 outbreaks

**DOI:** 10.3389/fpubh.2023.1202996

**Published:** 2023-07-13

**Authors:** Lichao Yang, Mengzhi Hu, Huatang Zeng, Wannian Liang, Jiming Zhu

**Affiliations:** ^1^Vanke School of Public Health, Tsinghua University, Beijing, China; ^2^Shenzhen Health Development Research and Data Management Center, Shenzhen, Guangdong, China; ^3^Institute for Healthy China, Tsinghua University, Beijing, China

**Keywords:** China-bound travel, COVID-19, non-pharmaceutical interventions, time-varying, health resource allocation

## Abstract

**Objectives:**

Non-pharmaceutical interventions (NPIs) implemented on China-bound travel have successfully mitigated cross-regional transmission of COVID-19 but made the country face ripple effects. Thus, adjusting these interventions to reduce interruptions to individuals’ daily life while minimizing transmission risk was urgent.

**Methods:**

An improved Susceptible-Infected-Recovered (SIR) model was built to evaluate the Delta variant’s epidemiological characteristics and the impact of NPIs. To explore the risk associated with inbound travelers and the occurrence of domestic traceable outbreaks, we developed an association parameter that combined inbound traveler counts with a time-varying initial value. In addition, multiple time-varying functions were used to model changes in the implementation of NPIs. Related parameters of functions were run by the MCSS method with 1,000 iterations to derive the probability distribution. Initial values, estimated parameters, and corresponding 95% CI were obtained. Reported existing symptomatic, suspected, and asymptomatic case counts were used as the training datasets. Reported cumulative recovered individual data were used to verify the reliability of relevant parameters. Lastly, we used the value of the ratio (Bias^2^/Variance) to verify the stability of the mathematical model, and the effects of the NPIs on the infected cases to analyze the sensitivity of input parameters.

**Results:**

The quantitative findings indicated that this improved model was highly compatible with publicly reported data collected from July 21 to August 30, 2021. The number of inbound travelers was associated with the occurrence of domestic outbreaks. A proportional relationship between the Delta variant incubation period and PCR test validity period was found. The model also predicted that restoration of pre-pandemic travel schedules while adhering to NPIs requirements would cause shortages in health resources. The maximum demand for hospital beds would reach 25,000/day, the volume of PCR tests would be 8,000/day, and the number of isolation rooms would reach 800,000/day within 30 days.

**Conclusion:**

With the pandemic approaching the end, reexamining it carefully helps better address future outbreaks. This predictive model has provided scientific evidence for NPIs’ effectiveness and quantifiable evidence of health resource allocation. It could guide the design of future epidemic prevention and control policies, and provide strategic recommendations on scarce health resource allocation.

## 1. Introduction

COVID-19 has created a global challenge that demands researchers, policymakers, and governments address multiple dimensions which go far beyond the implications of human health and well-being (1–4). Scientific evidence has indicated that non-pharmaceutical interventions (NPIs) are effective measures to contain a pandemic and ease pressures on healthcare systems (5–7). NPIs are actions, apart from getting vaccinated and taking medicine, that people can take to help slow the spread of illnesses, also known as mitigation strategies (8–10). It includes travel restrictions, contact tracing, PCR tests, measures in social distancing, personal protection, and quarantines (6, 11, 12). The implementation of such interventions while maintaining social stability is a challenge to all countries. As a country consisting of more than 1.4 billion or 18% of the world’s population, China’s high population density, high volume, speed, and non-locality of human mobility would provide perfect conditions for the virus to spread (13, 14). When highly transmissible Delta and Omicron variants resulted in massive surges in COVID-19 cases from December 2021 (15, 16), China saw the largest spike for the past 2 years, despite determinedly pursuing one of the world’s strictest virus elimination policies. When a local COVID-19 case occurred, mandatory interventions would be taken to cut off the transmission chain and terminate the outbreak in time to achieve maximum effectiveness with minimum cost. After years of exploration, such strategies’ implementation received remarkable results in containing regional cases (17, 18). However, it required extensive community involvement, government funding guarantees, application of new technology, motivation, and constraint mechanisms. Such a strategy created indefinable impacts on regional social development (19, 20). Thus, knowing how to maximize the advantages of strategy in outbreak control while avoiding damaging the development of the country was critically important. Due to the combined use of NPIs in the strategy, we decided to quantify the impact of different NPIs. Extensive research was conducted by using a time-varying modeling-informed approach and focusing on the following three interventions in this paper: inbound flight restrictions, PCR tests, and centralized quarantine measures.

Severe acute respiratory syndrome coronavirus-2 (SARS-CoV-2) viral spread patterns were shaped by the high volume of cross-country mobility networks (21). In response to the pandemic, China reduced inbound flight schedules from 10,000 per week in 2019 to 500 per week in recent years (22), and international arrivals were reduced from approximately 162.5 million in 2019 to 30.4 million in 2020 (23). In July 2021, the aviation authority updated requirements—passengers were required to complete a PCR test within 5 days of embarkation and provide negative test results before boarding, as the government tried to further reduce the risk of imported cases (24). However, from July 1 to July 31, 2021, 1,213 confirmed COVID-19 cases were reported across the country, compared with 1,893 cases in August and 1,264 cases in September (25). Although travel restrictions and PCR tests were proven as useful practices (26), the theoretical basis of those strategies and how to strategically align them with a country’s development was not studied.

There was a high level of agreement that the adoption of travel measures led to important changes in the dynamics of the early phases of the COVID-19 pandemic (27). Flight restrictions may have led to additional reductions in the number of exported and imported cases on the international scale, but such limitations (up to 90% of traffic) had only a modest effect unless combined with a 50% or higher reduction of transmission in the community (28). With the occurrence of domestic COVID-19 outbreaks, the association between international travel and the implementation of NPIs has not been identified. NPIs such as centralized hospitalization for mild and moderate patients could reduce disease transmissions and enhance protection for healthy and unhealthy individuals (29, 30). Nevertheless, the efficacy of mandatory isolation for international travelers at a designated place in a given period was not discussed. Research on Hong Kong-bound air passengers indicated that home quarantine was less effective than a centralized quarantine strategy initially but showed similar efficacy in the later phase (31). However, the effectiveness of self-isolation, transmission rate within the family cluster, related disease burden, and consumption of public health resources were not mentioned. According to a study published by United States Centers for Disease Control and Prevention, the transmission of SARS-CoV-2 among household members was common, and secondary infection rates were higher and occurred rapidly, with approximately 75% of infections identified within 5 days of the index patient’s illness onset (32). Substantial transmission occurred whether the index patient was an adult or a child, leaving no one healthy enough to help other family members.

Mathematical models and time series analyses have been widely used to study the pandemic and predict the trend. Researchers used a time-dependent SIR model to track the transmission and recovery rate at time t and presented less than 3% of one-day prediction errors (33). But the effects of NPIs were not discussed in the research. Another time-varying SIRD model was also developed to capture possible changes in the epidemic behavior, due for example to containment measures enforced by authorities or modifications of the epidemic characteristics and to the effect of advanced antiviral treatments in Italy (34). However, the research team did not take the interaction effects between containment measures and international travel bans into consideration. To infer more accurate parameter estimates and reduce uncertainties, scholars used real datasets of COVID-19 cases via an SEIR model with time-varying transmission and reporting rates to perform 1-week ahead predictions and generated more realistic interpretations (35). Despite that, this model was designed to predict the number of under-reported active cases not for NPIs evaluation, strategic planning, and resource allocation.

Thus, we would develop epidemiological models to simulate the domestic spread of SARS-CoV-2 sparked by passengers who had followed NPIs, such as inbound travel restrictions, quarantine measures, and PCR tests. However, the traditional epidemiological models fail to show the real-time implications of NPIs implementations, delayed symptoms, and test results. To present the time-varying effects, we developed a homogenous hybrid dynamic Susceptible-Infectious-Recovered (SIR) model to quantify such implications. The model can capture multiple data resources rather than a single dataset and generate a more robust estimation of the underlying dynamics of transmission from noisy data. Furthermore, it clearly described the synergistic effects of multiple interventions, such as face masks and social distancing. By combining an improved SIR model with four datasets collected from July 21 to August 30, 2021, we explored the sustained human-to-human transmission relationship between the inbound travelers and the domestic outbreaks under effective NPIs. Based on the simulation results, we formed a comprehensive model to quantify the impact of each NPIs and predicted the trend of future outbreaks based on the implementation of these NPIs. The goal was to ① explore the relationship between the imported cases and the development of the domestic epidemic, ② discuss how to adjust existing prevention and control strategies based on our findings, and ③ prepare sufficient health resources in advance while preventing health systems become overwhelmed. Moving forward, we would like to explore the balance point in epidemic prevention and international travel restrictions that could minimize the disruptions to social development.

## 2. Materials and methods

### 2.1. Model assumptions for consideration

The total population was 1,411,478,724 except for Hong Kong, Macau, Taiwan, and about 300,000 who are naturally immune (36).

- Assuming that the population is closed, meaning that there are no births and deaths. Population migration status change is considered during the study period, but they are dynamically stable, then
$$\begin{array}{l} S\left(t\right)+C\left(t\right)+Q\left(t\right)+I_{a}\left(t\right)+I_{s}\left(t\right)+\\ L_{i}\left(t\right)+L_{e}\left(t\right)+R\left(t\right)+D\left(t\right)=N\left(t\right)==N. \end{array}$$
- Assuming the population is homogeneously distributed and individuals mix uniformly.
- Assuming that the infectiousness of symptomatic and asymptomatic individuals is the same in a real-world scenario (37).
- Assuming that the recovered patients are negligible during the early stage of the pandemic and their presence will likely not affect the disease transmission (38–40).
- Assuming that symptomatic and asymptomatic cases will be moved into convalescence after rehabilitation due to COVID-19 immunity after infection.
- Assuming the effect of vaccines, average delays between symptom onset and test results are constant.
- Assuming all inboard and abroad travelers have performed the PCR tests, centralized quarantine, and completed treatments at designated hospitals.

### 2.2. A homogenous hybrid network-based model of SARS-CoV-2 transmission

The SIR model was used to model the spread of infectious diseases among a fixed population. This classic compartment model divided the population into susceptible (*S*), infected (*I*), and recovered (*R*) individuals and track the transitions of individuals among these states. It is a deterministic model of a homogeneous population with well-mixed interactions. Since China is continually updating its prevention and control measures, we extend the SIR modeling framework to nine classes: susceptible ( S ), carried ( C ), asymptomatic infected ( $I_{a}$ ), symptomatic infected ( $I_{s}$ ), recovered ( R ), quarantined ( Q ), dead ( D ), immigrated ( $L_{i}$ ), and emigrated ( $L_{e}$ ) to study the SARS-CoV-2 transmission on dynamic networks. Especially asymptomatic infected ( $I_{a}$ ) are individuals who show no symptoms but PCR test positive, and virus-carrier compartment ( C ) represents individuals who show no symptoms and PCR test negative but infectivity. Furthermore, quarantined ( Q ), immigrated ( $L_{i}$ ), and emigrated ( $L_{e}$ ) compartments are designed to analyze the effectiveness of NPIs, such as the inbound flight restriction, PCR test, and centralized quarantine.

**Figure 1:**
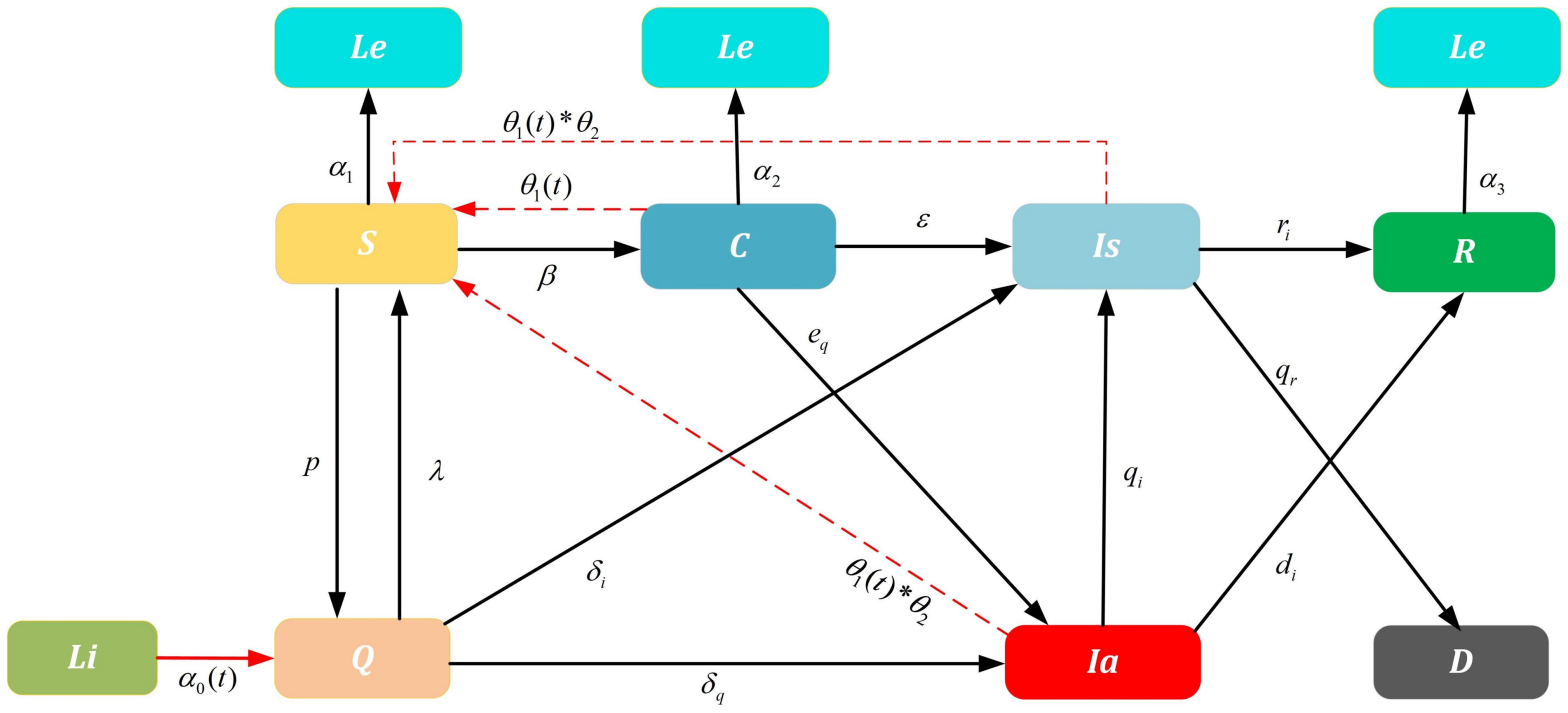
Improved SIR model on SARS-CoV-2 transmission. Dashed lines are influence parameters refer to a real-world scenario where the untraceable infections were reported. For example, untraceable infections that caused by contaminated cold-chain products *θ*_2_ and infections rate *θ*_1_(*t*) that trigger local outbreaks. Solid lines are transition probability of compartments. Parameters *α*_0_(*t*), *λ*, *δ*_*i*_ and *δ*_*q*_ are related to the NPIs implemented for China-bound travelers and *α*_1_, *α*_2_, and *α*_3_ are the outbound parameters; *r*_*i*_,*d*_*i*_ are the recovery rate and *q*_*r*_ is the death rate; *p*, *β*, *ε*, *e*_*q*_, *q*_*i*_ are the transition probability. Furthermore, the arrows represent the direction of transition/influence between compartments. With above, the initial values and detailed values are presented in Tables 1, 2.

In the system of improved SIR model (Figure 1), $\alpha_{0}\left(t\right)$ represents the percentage of inbound passengers. They are required to stay in a designated place for X days upon arrival and receive closed-loop care. A portion λ of Q will move to S , a portion $\delta_{i}$ of Q will move to $I_{s}$ , and a portion $\delta_{q}$ of Q will move to $I_{a}$ . Once they entered into the susceptible group S , there is a risk ratio β of S to move some of them into C and diagnosed as $I_{s}$ or $I_{a}$ by the transfer rate of ε and $e_{q}$ respectively. In addition, a portion p of S determined by close contact and sub-close contact tracing will move to quarantined Q . In the meantime, a portion of $q_{i}$ and $q_{r}$ represent the $I_{a}$ will move to $I_{s}$ and R. With the above, since population fraction in compartments $S,C,Q,I_{a},I_{s},L_{i},L_{e},R,D$ varies with time t (in days), we assume *S*(*t*) + *C*(*t*) + *Q*(*t*) + *I*_*a*_(*t*) + *I*_*s*_(*t*) + *L*_*i*_(*t*) + *L*_*e*_(*t*) + *R*(*t*) + *D*(*t*) = *N*(*t*)==*N*, the following kinetic equation is obtained. Initial values, conditions, and descriptions are presented in Table 1.


$$\frac{dS}{dt}=-\left(p+\beta \right)S\left(\theta_{1}\left(t\right)C+\theta_{1}\left(t\right)*\theta_{2}I_{a}+\theta_{1}\left(t\right)*\theta_{2}I_{s}\right)-\alpha_{1}S+\lambda Q$$

$$\frac{dC}{dt}=\beta S\left(\theta_{1}\left(t\right)C+\theta_{1}\left(t\right)*\theta_{2}I_{a}+\theta_{1}\left(t\right)*\theta_{2}I_{s}\right)-\left(\alpha_{2}+\varepsilon +e_{q}\right)C$$

$$\frac{dQ}{dt}=pS\left(\theta_{1}\left(t\right)C+\theta_{1}\left(t\right)*\theta_{2}I_{a}+\theta_{1}\left(t\right)*\theta_{2}I_{s}\right)+\alpha_{0}\left(t\right)L_{i}-\left[\lambda +\delta_{q}+\delta_{i}\right]Q$$

$$\frac{dI_{a}}{dt}=\delta_{q}Q+e_{q}C-\left(q_{i}+q_{r}\right)I_{a}$$

$$\frac{dI_{s}}{dt}=\varepsilon C+\delta_{i}Q+q_{i}I_{a}-\left(r_{i}+d_{i}\right)I_{s}$$

$$\frac{dR}{dt}=r_{i}I_{s}+q_{r}I_{a}-\alpha_{3}R$$

$$\frac{dD}{dt}=d_{i}I_{s}$$

$$\frac{dL_{e}}{dt}=\alpha_{1}S+\alpha_{2}C+\alpha_{3}R$$

$$\frac{dL_{i}}{dt}=-\alpha_{0}\left(t\right)L_{i}$$

**Table 1:** Initial conditions description for models.

Parameter	Meaning	Value	Source
p	Isolation rate of susceptible class	0.00000015	Ref (40)
$\alpha_{1}$	Exit rate of the susceptible class	$0.00000011*x_{1}$	Reported data
$\alpha_{2}$	Exit rate of the carried class	$0.0001*x_{1}$	Reported data
$\alpha_{3}$	Exit rate of the recovered class	$0.0000057*x_{1}$	Reported data
$\theta_{1}\left(t\right)$	Relative transmission strength of carried class to the susceptible class	-	See formula (8)
$\theta_{2}$	The probability of local outbreak	0.1194	See formula (9)
β	The transmission parameter of Delta variant	0.000000001	Ref (41)
ε	Transfer rate of carried class to the symptomatic class	0.1515 = 1/4.4*2/3	Ref (42)
$r_{i}$	Recovery rate of the symptomatic infected individuals	[0.0357,0.0714]	Reported data
$d_{i}$	Death rate due to infection	0	Reported data
$e_{q}$	Transfer rate of concentration quarantine susceptible individuals to the symptomatic infected class	0.0758 = 1/4.4*1/3	Ref (42)
$\alpha_{0}\left(t\right)$	Entry rate from foreign region to the mainland China	-	See formula (1)
τ(t)	The initial weigh value of effective duration of PCR test	-	See formula (2)
ϑ(t)	The initial weigh value of number of immigration flights	-	See formula (3)
λ	Release rate of concentration quarantine susceptible individuals to the susceptible class	-	See formula (5)
$\delta_{i}$	Transfer rate of concentration quarantine susceptible individuals to the symptomatic infected class	-	See formula (6)
$\delta_{q}$	Transfer rate of concentration quarantine susceptible individuals to the asymptomatic infected class	-	See formula (7)
$q_{r}$	Recovery rate of the asymptomatic infected individuals	[0.0357,0.0714]	Reported data
S(0)	Initial value of susceptible individuals in the free environment	1.41007756*e*^+09^−*L*_*i*_(*t*)	Reported data
C(0)	Initial value of existing carried cases	6	Reported data
$I_{s}\left(0\right)$	Initial value of existing symptomatic cases	638	Reported data
R(0)	Initial value of cumulative recovered individuals	87,140	Reported data
D(0)	Initial value of cumulative deaths	4,346	Reported data
Q(0)	Initial value of existing suspected cases	8,577	Reported data
$I_{a}\left(0\right)$	Initial value of existing asymptomatic cases	456	Reported data
$L_{i}\left(0\right)$	Initial value of cumulative immigration	$x_{1}*211*41/0.68$	Reported data
$L_{e}\left(0\right)$	Initial value of existing emigration	0	Reported data
N	Total population in the mainland China	1,411,478,724	Reported data
$x_{0}$	The effective duration of PCR test	[2,3,4,5,6,7,8,9,10,11,12,13,14]	Reported data
$x_{1}$	The number of immigration flights	[20,40,60,79,100,120,140,160,180,320,640,1,000,1,366]	Reported data
$x_{2}$	The strengths of centralized isolation and quarantine	[10,14,17,21,24,28,31,35,38,42,45,49,52]	Reported data
γ	The weight parameter of incubation period	-	See formula (4)

### 2.3. The designed functions are related to fitted parameters

Multipronged interventions have considerable positive effects on minimizing the spread of outbreaks, decreasing the reproduction number, and reducing total infections. To further clarify the mechanism of interventions and additive effect on epidemic, parameters $\alpha_{0}\left(t\right)$ , λ , $\delta_{i}$ , and $\delta_{q}$ related to the NPIs implemented for China travelers are constructed in the improved SIR model. Especially $\alpha_{0}\left(t\right)$ is a comprehensive parameter determined by the parameter τ(t) related to interventions PCR test and the parameter ϑ(t) related to inbound flight restrictions. The parameters $\lambda ,\delta_{i}$ , and $\delta_{q}$ are dependent on centralized quarantine measures. Those four dependent variables are mainly changed by the independent variables, i.e., $x_{0}$ , $x_{1}$ , $x_{2}$ , and γ . $x_{0}\;$ represents the validity period of the PCR test, $x_{1}$ is the number of international flights, $x_{2}$ is the strength of the centralized quarantine measure, γ is the weight parameter related to the incubation period of SARS-CoV-2.

$\alpha_{0}\left(t\right)$ as the main explanatory variable, signifies the proportion of the population migrating to China from other countries. We have modeled the population entry rate via the contribution of the validity period of the PCR test and the restrictions on international flights according to the characteristic of immigration by actual data tracing, shown as the formula (1):


(1) $$\alpha_{0}\left(t\right)=\tau \left(t\right)+\vartheta \left(t\right)$$

To simulate the number of international flights, we set parameters τ(t) and ϑ(t) varying with time t . τ(t) represents the contribution of the effective duration of the PCR test and ϑ(t) represents the contribution of the number of inbound flights on population entry rate at time t . Then we find τ(t) is linear to the weight parameter $e_{1}\;$ (43), and the weight portion is e times the reciprocal relationship with the number of inbound flights $x_{1}$ and is logarithmic with the effective duration of nucleic acid testing $x_{0}\;$ (44). ϑ(t) is linear to the weight parameter $l_{1}$ , and the weight portion is l times reciprocal relationship with the effective duration of the PCR test by fitting to the data (45):

(2) $$\begin{array}{c} \tau \left(t\right)=e/x_{1}*\log\left(\gamma *x_{0}\right)+e_{1}t \end{array}$$

(3) $$\begin{array}{c} \vartheta \left(t\right)=l/\log\left(x_{1}\right)+l_{1}t \end{array}$$

γ is the weight parameter only affected by the effective duration of the PCR test $x_{0}$ . After we draw a curve of best fit, we find the effects of PCR test validity period setting are in line with the logarithmic function. This means the virus incubation period could influence the test validity period (46). When the test validity period is shorter than the incubation period, the effect of the validity period of the PCR test conforms to the significant variation part of the logarithmic function, so set γ=1 . If the test validity period is longer than the incubation period, the effect of the validity period of the PCR test conforms to the gently part of the logarithmic function, so set $\gamma =100000^{x_{0}}$ .


$$\gamma =1,if\;x_{0}\leq thelengthoftheaverageincubationperiod$$

(4) $$\begin{array}{c} \gamma =100000^{x_{0}},if\;x_{0}>thelengthoftheaverageincubationperiod \end{array}$$

The parameter λ is the release ratio at the end of the quarantine, which follows an exponential distribution with parameters c1 and c (47):


(5) $$\begin{array}{c} \lambda =c*e^{-c_{1}*x_{2}} \end{array}$$

The parameter $\delta_{i}$ is the probability of the quarantine measure to the symptomatic infectious individuals, and the parameter $\delta_{q}$ is the probability of the quarantine measure to the asymptomatic infectious individuals (48). Additionally, $\Delta_{0}$ , $\rho_{0}$ , $\eta_{0}$ , $\Delta_{1}$ , $\rho_{1}$ , and $\eta_{1}$ are all the fitting parameters, we also derive the 95% confidence interval (CI), which is shown in Table 2:


(6) $$\begin{array}{c} \delta_{i}=\Delta_{0}+\rho_{0}\log\left(\eta_{0}*x_{2}\right) \end{array}$$

(7) $$\begin{array}{c} \delta_{q}=\Delta_{1}+\rho_{1}\log\left(\eta_{1}*x_{2}\right) \end{array}$$

**Table 2:** Estimated parameters description for models.

Parameter	Meaning	95%CI	Value	Source
$\Delta_{0}$	Minimum conversion rate	(0.00000881, 0.000011)	0.00001	Estimated
$\rho_{0}$	Adjustment coefficient	(0.00021, 0.00024)	0.00023	Estimated
$\eta_{0}$	Adjustment coefficient	(990,1,011)	1,000	Estimated
$\Delta_{1}$	Minimum conversion rate	(0.0000009,0.0000011)	0.000001	Estimated
$\rho_{1}$	Adjustment coefficient	(0.00015, 0.00016)	0.00016	Estimated
$\eta_{1}$	Adjustment coefficient	(2.89, 3.11)	3	Estimated
$q_{i}$	Transfer rate	(0.008, 0.011)	0.01	Estimated
c	Weight parameter of controlling increasing rate	(0.66, 0.72)	0.68	Estimated
$c_{1}$	Exponential decline rate	(0.00008,0.00012)	0.0001	Estimated
e	Logarithmic increment rate	(0.009,0.011)	0.01	Estimated
l	Logarithmic increment rate	(0.0235, 0.0265)	0.025	Estimated
$e_{1}$	Linear increasing rate	(0.00214,0.00216)	0.00216	Estimated
$l_{1}$	Linear increasing rate	(0.000018,0.0000219)	0.00002	Estimated

In the context of infectious disease control, curtailing interactions between infected and susceptible populations, reducing the infectiousness of symptomatic patients, reducing the susceptibility of susceptible individuals, and scaling up such intervention coverage to accommodate rapid increases in the number of suspected cases are well-known strategies for minimizing pandemic spread (49). China has adopted measures conforming to China’s conditions based on the strategic theory, i.e., local management. When an outbreak occurs, a local management strategy will be implemented in that particular city. To model the local management policy concretely, dynamic parameter $\theta_{1}\left(t\right)$ varying with time is introduced to the improved model. The parameter is determined by the number of cities with infected cases and the population of each city. To enhance the generation ability of the model, we set the city size equal to 4,000,000 residents (50). Since the centralized quarantine strategy of inbound flights is managed in a closed loop, and researches show the majority of domestic outbreaks were caused by contaminated imported cold-chain food (51, 52) which was less traceable, we set $\theta_{2}$ as the probability of infection caused by cold-chain propagation.


(8) $$\begin{array}{c} \theta_{1}\left(t\right)=\frac{Thepopulationsizeofoutbreakcity}{N} \end{array}$$

(9) $$\begin{array}{c} \theta_{2}=\frac{Thefrequencyofoutbreakscaused\;by\;cold-chain}{Thefrequencyoftotaloutbreaks} \end{array}$$

### 2.4. Data resource

July 21, 2021, was set as the starting date of this study. The initial value of S(0) was collected from the Seventh National Population Census. The initial values of existing symptomatic cases $I_{s}\left(0\right)$ , existing asymptomatic cases $I_{a}\left(0\right)$ , existing suspected cases Q(0) , cumulative recovered individuals R(0) , and cumulative deaths D(0) were captured from July 21, 2021, based on the National Health Commission of China reports. $L_{e}\left(0\right)$ and $L_{i}\left(0\right)$ were collected from VariFlight since July 21, 2021. Since the incubation period is around 4 days, the existing virus-carried cases C(0) were set to equal to the new domestic case count after (0 + 4) days, i.e., July 25, 2021. Based on VariFlight data and travel requirements, all international flights’ capacity were set to equal to 50% of the original capacity. For better versatility, the average population for medium-sized cities in China was set as 4,000,000 (53).

### 2.5. Parameters setting and parameters estimation

According to VariFlight, there were an average of 16,707 inbound immigrants and 12,310 outbound emigrates per day. Deidentified aggregated data collected from July 21, 2021, to August 30, 2021, was used to fit the inbound parameter $\alpha_{0}\left(t\right)$ , the outbound parameters $\alpha_{1},\alpha_{2}$ , and $\alpha_{3}\;$ (54). To study the impact of the scenario with the normal inbound flights on the domestic outbreaks and economic development, we collected the historical data from July 1 to 14, 2019, to simulate the future flow trend of inbound travelers, observe the development trend of COVID-19 and summarize recommendations.

Theoretically, without considering the epidemiological characteristics of SARS-CoV-2, this generic improved SIR model could provide estimation with the above parameters ( $p,\beta ,\varepsilon ,r_{i},d_{i},e_{q},q_{r}$ ). Parameters p and β were defined via reference (40). However, as the Delta variant continued to mutate, the early transmission rate β was lower than the current variation (Table 3). In addition, the average incubation period of the Delta virus was about 4.4 days and about two-thirds of those infectious cases were symptomatic (55), corresponding to $\varepsilon +e_{q}=1$ , as a result, we set the transfer rate ε as 1/4.4*2/3. Furthermore, according to the study report (53), the average recovery period was between 14 and 28 days, thus we set $r_{i}$ and $q_{r}$ equal to (*1/28*, *1/14*). Lastly, historical data has shown zero deaths during the selected period, so $d_{i}$ was set as zero.

**Table 3:** SIR model stability analysis.

Datasets	Training datasets			Testing dataset
	Existing symptomatic cases	Existing suspected cases	Existing asymptomatic cases	Cumulative recovered individuals
${\mathrm{Bias}}^{2}$	177701.8000	205784670.7000	1724.8290	327447.3000
Variance	210671.1451	165107018.8000	3089.3700	648821.8800
${\mathrm{Bias}}^{2}/\mathrm{Variance}$	0.8435	1.2463	0.5583	0.5046

To investigate how NPIs implementation impacts the outbreak duration or the turning point, the logic parameters ( $\Delta_{0}$ , $\rho_{0}$ , $\eta_{0}$ , $\Delta_{1}$ , $\rho_{1}$ , $\eta_{1}$ , e , l , $e_{1}$ , $l_{1}$ , $q_{i}$ , c , $c_{1}$ ) associated with fitting functions were estimated by Monte Carlo Stochastic Simulation (MCSS) approach. To get the probability distribution for variables related to population behaviors, a large number of simulation repetitions were needed to stabilize the frequency distributions. Parameters were randomly generated within a range equal to their best fit to the observed data or literature via efficient Python software, then we ran the MCSS method with 1,000 iterations to derive the probability distribution of those variables. Finally, we obtained the initial values and estimated parameters of the model, and listed parameters, initial values, as well as corresponding 95% CI in Table 2.

We further compared the prediction results with three training datasets to determine their final parameters solution aiming to minimize RMSE. To verify the validation of the SIR model and estimated parameters, we compared the model with the testing dataset. Predictive results indicated that the estimated values were in very good agreement with real reported data and that the estimated parameter values can be used to predict the future development trend of COVID-19 in mainland China.

## 3. Results

### 3.1. Model verification of reliability, stability, and sensitivity

**Figure 2:**
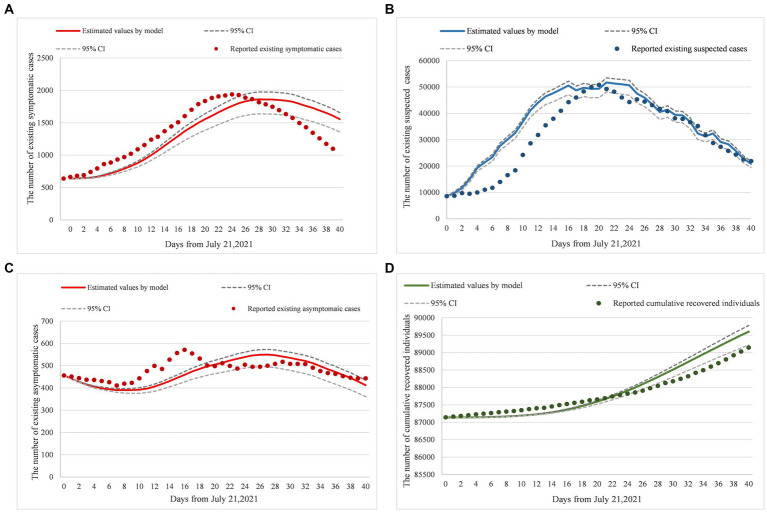
**(A–D)** Model fitting and real-world data comparison. Panels **(A–D)** were the verification results of model parameters inputting, compared existing symptomatic cases, existing suspected cases, existing asymptomatic cases, and cumulative recovered individual datasets, from July 21, 2021 to August 30, 2021 based on the National Health Commission of China reports. Additionally, dotted lines were the 95%CI of prediction results, solid lines were the prediction results by model inputting, and original points were the statistical data from the National Health Commission of China reports. Moreover, the RMSE of **(A)** is equal to 194.35; the RMSE of **(B)** is equal to 6733.59; the RMSE of **(C)** is equal to 46.54; and the RMSE of **(D)** is equal to 283.57.

Figures 2A–D were simulated based on existing symptomatic, suspected, and asymptomatic cases and cumulative recovered individual datasets, reported by the National Health Commission of China from July 21, 2021, to August 30, 2021. The reported existing cases were set as training datasets to generate (Figures 2A–C). Reported cumulative recovered individuals were used as a testing dataset to generate (Figure 2D). To verify the model’s reliability, root mean square error (RMSE) was adopted to cross-validate the predicted results and the real-world results. Since a smaller RMSE result refers to a better fitting result, by putting the weight vector quantity (1,0.1,1) to training datasets to reach a goal of minimum RMSE, we obtained the optimal parameters solution. Finally, for reliability verification, the optimal parameters were assigned to the target model to obtain the predicted results and compare it with the trend of cumulative recovered individuals.

To verify the model’s stability while generating the best model fitting result, we identified the equilibrium point between variance and bias, and set the value of ratio (Bias^2^/Variance) in the interval [0.5,1.3], based on bias-variance dilemma theory (Table 3).

The sensitivity of NPIs on infected cases was tested in this section. Since the amount of three intervention combinations was 2,197, it was unrealistic to observe the effect of simultaneous changes on infected cases. In this paper, the changing influence of each NPIs on infected cases was observed while the other two NPIs maintain normal. Especially, Based on July 21, 2021, to August 30, 2021, NPIs requirements ( $x_{0}=2,x_{1}=79,x_{2}=17$ ), We completed sensitivity analysis on each travel-related intervention with input parameters, for example, the parameters e ， $e_{1}$ of validity period setting of PCR test, the parameters l , $l_{1}$ of the control of inbound flights, and the parameters c , $c_{1}$ , $\Delta_{0}$ , $\rho_{0}$ , $\eta_{0}$ , $\Delta_{1}$ , $\rho_{1}$ , and $\eta_{1}$ of the strength of centralized quarantine. To quantify the parameter sensitivity of each intervention, we set the number of infected cases caused by current travel interventions as N*. Then the intensity of each intervention was set to vary around its mean 20%, to derive *N*_*i*_. We calculated the relative error of input parameters of each intervention according to the formula [*abs*(N^*^−*N*_*i*_)/N^*^], as listed here (Table 4). We could observe that the input parameters sensitivity of the validity period setting of the PCR test was the highest, and the sensitivity of the control of inbound flights was the lowest. Thus, the results showed the input parameters of the PCR test were more stable than the other two types of input parameters.

**Table 4:** SIR model sensitivity analysis.

The window of related parameters/multiple proportions	Relative error of the validity period setting of PCR test	Relative error of the control of inbound flights	Relative error of the strength of the centralized quarantine
0.8	0.0794	0.0133	0.0380
1	0	0	0
1.2	0.0692	0.0137	0.0299

### 3.2. Demand for health resources

**Figure 3:**
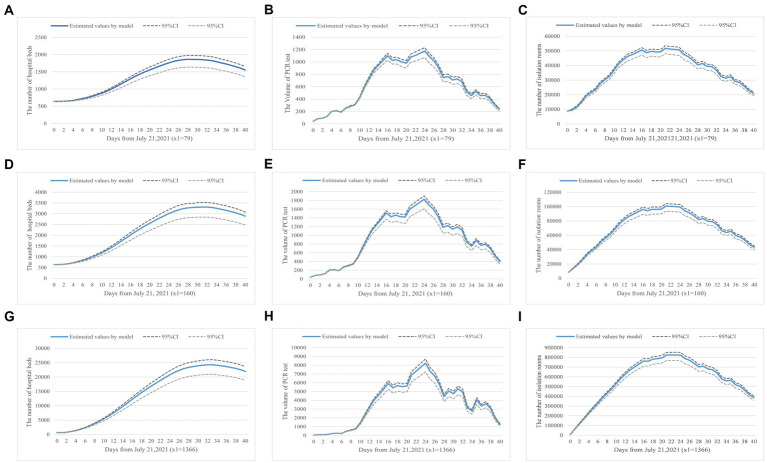
**(A–I)** Health resource demand prediction based on number of inbound flights. Panels **(A–C)** show the demand for hospital beds, PCR tests, and isolation rooms in a real-world scenario where the daily inbound flight is equal to 79. Panels **(D–F)** simulate the changes when inbound flights are 160, a scenario where the current requirements have been slightly lifted. Panels **(G–I)** present results when the number of inbound flights is 1,366, a scenario with no inbound flight restrictions.

The prevalence of COVID-19 worldwide will increase the risk of local transmission. Our model has described a scenario on how to allocate health resources in preparation for possible outbreaks when international flights have been reduced from 1,366 to 79. Figure 3 showed the predictive demand for hospital beds, PCR test volume, and centralized isolation rooms.

Firstly, Figures 3A,D,G showed how the number of international flights impacts hospital bed demand. We stipulated the number of beds in use was configured to be equal to the number of infected individuals to visualize hospital bed occupancy based on the China CDC’s requirements (56).

Figures 3B,E,H simulated the demand for PCR tests, which was achieved by the product of the obtained number of virus carriers and their highest transmission coefficient. Lastly, Figures 3C,F,I indicated the isolation rooms demand varies with the number of inbound flights. Based on the current quarantine requirements, one person per private hotel room, we could configure the isolation rooms in unit proportion with the isolated population.

Based on the analysis, we found that when the number of international flights was doubled (*x*_1_ = 160), the number of hospital beds in use would increase by 83%, the PCR test volume would increase by 44%, and the number of isolation rooms in need was doubled. The results showed that the growth in the number of international flights had the greatest impact on isolation room demand. When the number of international flights increased from 79 to 1,366, the demand for hospital beds raised to 25,000/day, the PCR test volume was up to about 8,000/day, and 800,000/day isolation rooms within 30 days were in need in preventing the spread of the epidemic. Our simulation results indicated that, under those epidemic prevention and control strategies, China was not ready to fully resume pre-pandemic international travels due to excessive demand for health resources. Additionally, the prevalence of COVID-19 in the surrounding countries would increase the probability of a sizable domestic outbreak. To prevent excess demand for health resources, the implementation of an aggressive disease prevention and control strategy was recommended.

As the virus continues to evolve, China is likely to readjust its preventive policies, we will discuss how these future modifications would impact the spread of disease and demand for health resources in the follow-up study.

### 3.3. Effectiveness of NPIs and risk warning of domestic outbreaks

Our retrospective model has indicated that NPIs on travel requirements have successfully contained the spread of the virus. In this section, we will discuss the control of the number of inbound flights, the validity period setting of PCR tests, and the strength of centralized quarantine. By observing and analyzing changes in the number of infected cases and level of intervention implementation, the result will show the effectiveness of NPIs and risk warning of future domestic outbreaks.

#### 3.3.1. The validity period setting of PCR test

**Figure 4:**
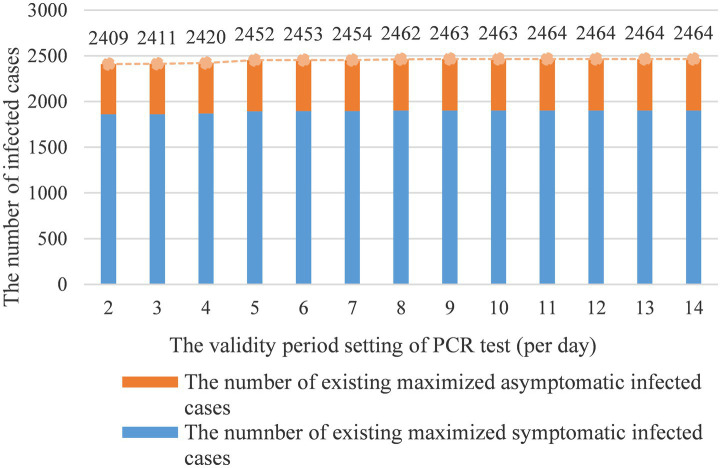
The validity period setting of PCR test vs. infected cases count.

Figure 4 shows that with the increase of validity period setting of PCR test, the number of symptomatic and asymptomatic infected cases will continue to grow until converging to a stable state presenting no effect of the intervention. Figure 4 shows there are no major fluctuations in the number of infected cases when the validity period of the PCR test is in a 4-day window. However, when we adjust the validity period to 7 days and more, the number of infected cases will be in a stable state. Our results show it is necessary and urgent to set a PCR test time requirement before travelers’ arrival. Secondly, the simulation shows that the validity period of the PCR test is closely related to the incubation period of the Delta variant, thus, the test validity period is suggested to be set within 4 days. To maximize impacts, the validity period should not exceed 7 days.

#### 3.3.2. The control of inbound flights

**Figure 5:**
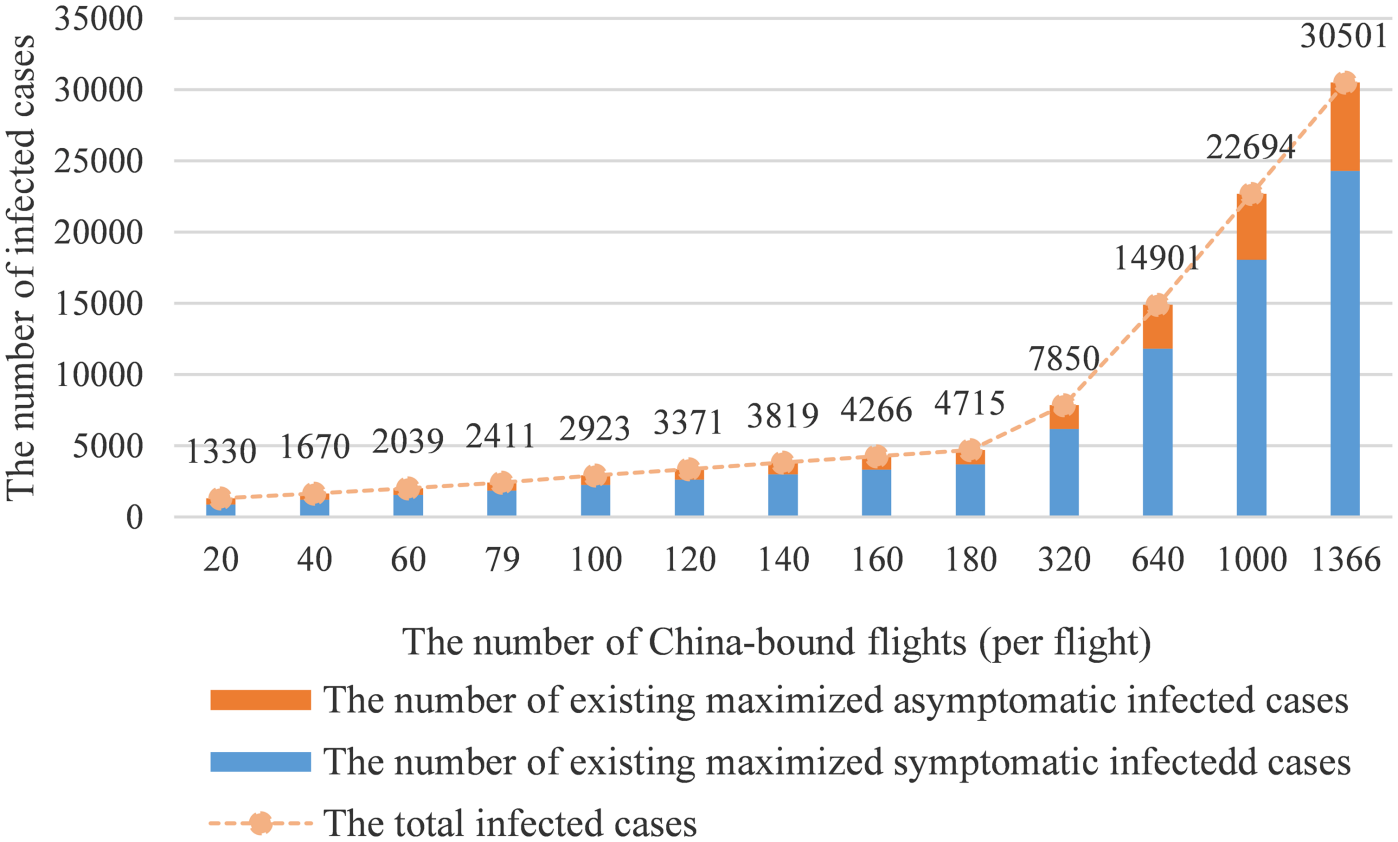
The number of inbound flights vs. infected cases count.

Figure 5 reveals the relationship between the number of inbound flights and the infected case count. As the number of international flights increases, the number of infected cases would grow exponentially. In this part, we adjust the inbound flight number from 20 to 180 with arithmetic progression and proportional sequence. The simulation results show when the inbound flight number equals 79 per day, there will be approximately 2,411 infected cases. When the inbound flight number exceeds 180 per day, the number of infected cases would rise to 4,715. When the number of inbound flights equals 1,366 per day, the daily infected cases would achieve 30,501. These results supported the following conclusions: first, the simulation results show that the change in flight numbers has a greater impact than other interventions, thus, limiting the number of inbound flights is the most effective intervention in preventing local transmissions. As a result, the adjustment of the intervention should be considered carefully, because the change in 3–4 infected cases count could trigger a local outbreak under the current severe international situation (47).

#### 3.3.3. The strength of centralized quarantine

**Figure 6:**
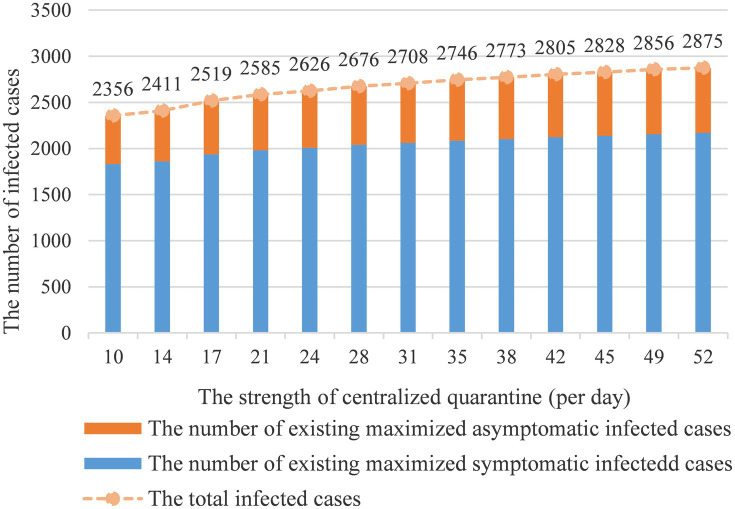
The strength of centralized quarantine vs. infected cases count.

Figure 6 shows how centralized quarantine influences the number of infected cases. With the extension of the quarantine period, the number of infected cases will continue to grow. It can be observed that the impact of the intervention is still remarkable within threshold 35 on preventing the spread of the epidemic and the number of infected cases is converging to a stable situation when exceeding threshold 35. The model also indicates that 17 days of centralized quarantine would effectively prevent disease spread. The quarantine benefit will diminish after 17 days benchmark and reach a stable state after 35 days.

#### 3.3.4. Comprehensive review of all interventions

**Figure 7:**
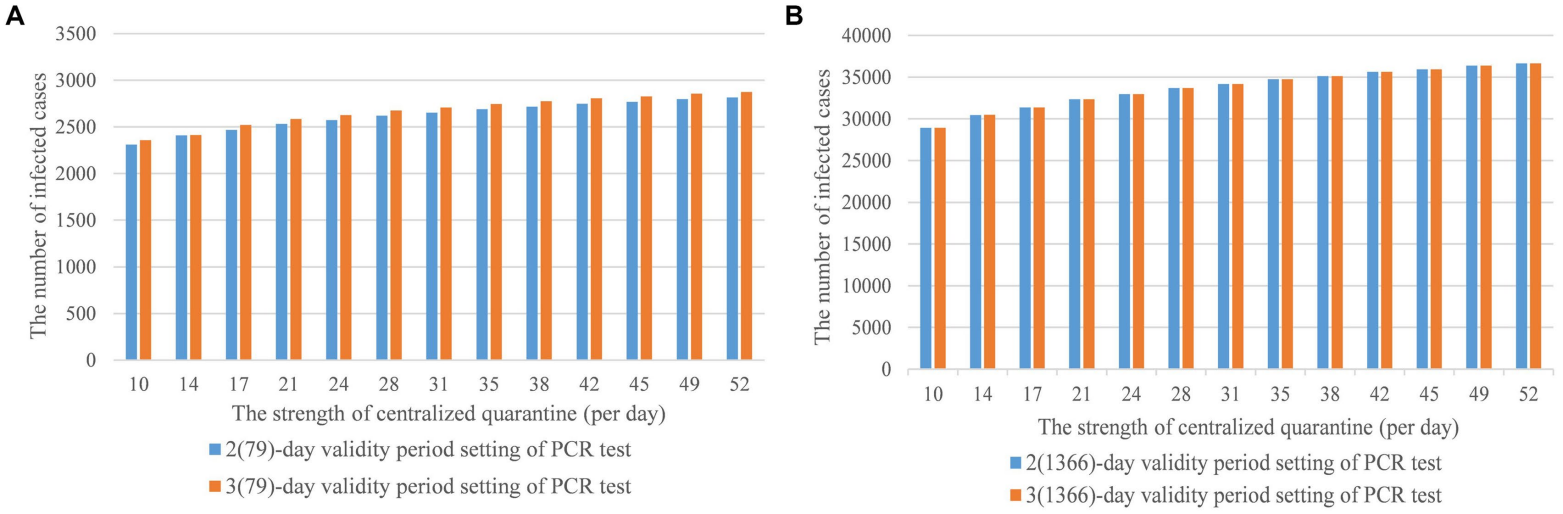
**(A,B)** The strength of centralized quarantine and the validity period setting of PCR test.

Figure 7A simulated the interaction of the strength of centralized quarantine and the validity period setting of PCR test on the development of domestic epidemic in the current number of inbound flights scenario. Under two scenarios, where the number of restricted inbound flights was equal to 79 and the number of recovered normal inbound flights was 1,366, the 3-day of validity period setting would cause more local infected cases compared with the 2-day setting, especially in the recovered normal inbound flights scenario in Figure 7B. To quantify the difference in the infected cases between 2(79)- and 3(79)-day in the restricted scenario, we used RMSE to measure the gap, deriving about 53.431. For the small difference between 2(1366) and 3(1366)-day in the recovered scenario, RMSE is 1.0377. Thus, the 2(79)-day PCR test was recommended for the flight-restricted scenario and the 2(1,366) or 3(1,366)-day test was recommended for a normal schedule.

## 4. Discussion

### 4.1. Application of the improved SIR model from a macro perspective

Studies performed in the United Kingdom and the United States indicated that the effectiveness of any single NPIs was likely to be limited, combining multiple interventions was worthy of further study (57). Scholars also indicated that the effectiveness of travel bans in reducing the spread of infectious diseases, and the relative effectiveness of NPIs for controlling the pandemic has gone largely unstudied (58, 59). Therefore, our proposed model played a significant role in estimating the combined effects of NPIs implementation and predicting the demand for isolation rooms, PCR test volume, and hospital beds. The results could provide scientific guidance for nationwide strategic planning and policy implementation and also bridge the theoretical gaps between international travel controls and related effectiveness of the NPIs.

On one hand, we analyzed how inbound flights would impact the distribution of health resources in response to a possible local outbreak. The model quantified the impact of local virus carriers that supported PCR testing arrangements for community screening. The number of infected cases and quarantined population could support the allocation of hospital beds and the configuration of isolation rooms. Thus, we recommended that the government should restore inbound flight numbers appropriately with sufficient medical supply in response to the increase in daily infected cases.

On the other hand, our model and related results provided scientific evidence that supported the design and implementation of existing interventions. The results indicated that comprehensive interventions of a two-day PCR test, 79 inbound flights per day, and 17 days of centralized quarantine were effective in stabilizing domestic disease transmission. In addition, the modeling effort also provided theoretical advice for future adjustment. When the epidemic prevention and control goal is to treat and monitor the health status of all infected individuals, limiting the inbound flight number to a small scale is recommended. When the priority is to treat severe and critical cases in hospitals and monitor the health status of individuals who have mild or no symptoms at home, resuming a regular inbound flight schedule is recommended.

### 4.2. Application of the improved SIR model from a micro perspective

The risk estimation of COVID-19 importation can be applied to identify the effectiveness of travel-related control measures (60). However, the connection between imported cases and local outbreaks was not studied. In our model, parameters $\theta_{1}$ and $\theta_{2}$ were key factors to understand and mitigate domestic outbreak risks, and also represented mathematical logic interaction associated with the domestic outbreak and global pandemic status. Going further, the current improved SIR model provided more heuristic thinking for constructing new models for domestic outbreaks affected by various factors.

### 4.3. Application of the improved SIR model at other variants of SARS-CoV-2, such as omicron

The new variant SARS-CoV-2 Omicron demonstrated partial vaccine escape and high transmissibility, with early reports indicating lower severity of infection (47) and reduced risk of hospitalization (61) than pre-existed variants. We would like to extend the delta-focused simulation model and related control strategy parameters to Omicron and discuss the applicability and sustainability of the continued implementation of such strategies in combating the new variants in our future research.

### 4.4. Limitations

Our study has several limitations. First, our model did not consider individuals’ preventative behaviors. Secondly, we only considered the nationwide prevention strategies and did not dive into detailed strategies enacted at the province and city levels. To minimize such impacts, we adopted reasonable assumptions about epidemiological parameters and aspects of human behaviors that contributed to disease transmission. Although the results showed that our conclusions were remarkably robust, this model was highly sensitive to the quality of input parameters. Thus, we cautiously selected parameters and values based on literature research results and research data. In the proposed homogeneous hybrid model, the population and individuals were distributed and mixed homogeneously and uniformly. Disturbances, such as economic status, political environments, living environments, cultural influences, etc. remained the same. In the meantime, the transmission coefficient, and average delay between symptom onset and test results were constant, and the effect analysis of vaccines, the reporting delays, and testing delays were not captured, which would lead to the requiring hospitalization or developing severe COVID-19 stochastic by nature. Since the purposed model was set to make conservative predictions, when a new variant presents different severity, infectiousness, and immune escape features, we need to convert the purposed model with updated parameters and generate up-to-date predictions. Finally, the model neglected the stochastic effects at low-case numbers. When imported infections were reported, especially when testing was required, having or not a population-scale outbreak was a matter of probabilities; differential equation models cannot capture this accurately. Furthermore, for a disease like COVID-19 with such an over-dispersed individual variation of infectiousness (62), outbreaks were likely to die out if very few cases were introduced (63).

### 4.5. Conclusion

Our finding indicated that restriction on inbound flight numbers played a key role in preventing and controlling the epidemic, but the combined use of other NPIs would maximize the effect in preventing additional transmission. Centralized quarantine days should be set in between 17 to 35 days for the Delta variant. The validity period of the PCR test was related to the disease incubation period, and the valid time should be less than 7 days. In addition, when the disease incubated, the PCR test period did not have a significant impact on epidemic control. More importantly, the model estimated that if recovering the pre-pandemic inbound travel strategy in 2019, the number of hospital beds would reach 25,000 per day, the volume of PCR tests would be 8,000 per day, and the isolation capacity would be nearly 800,000 per day within 30 days to maintain the same achievement of preventing outbreaks. All in all, our improved model, which can robustly generate scenarios, will help understand the tradeoffs between different strategies, and further guide the health resources preparation and allocation.

## Data availability statement

The original contributions presented in the study are included in the article/supplementary material, further inquiries can be directed to the corresponding authors.

## Author contributions

LY, MH, HZ, WL, and JZ developed this project and created the manuscript concept. LY designed the model, interpreted the results, and wrote and edited the manuscript. MH contributed to the literature research, data collection, manuscript writing, and editing. All authors contributed to the article and approved the submitted version.

## Funding

This was funded by the National Natural Science Foundation of China (grant number 72091514); the Sanming Project of Medicine in Shenzhen (grant number 20212001132); and the Bill & Melinda Gates Foundation (grant number INV-018302).

## Conflict of interest

The authors declare that the research was conducted in the absence of any commercial or financial relationships that could be construed as a potential conflict of interest.

## Publisher’s note

All claims expressed in this article are solely those of the authors and do not necessarily represent those of their affiliated organizations, or those of the publisher, the editors and the reviewers. Any product that may be evaluated in this article, or claim that may be made by its manufacturer, is not guaranteed or endorsed by the publisher.

## References

[1] Lambert H Gupte J Fletcher H Hammond L Lowe N Pelling M. COVID-19 as a global challenge: towards an inclusive and sustainable future. Lancet Planet Health. (2020) 4:e312–4. doi: 10.1016/S2542-5196(20)30168-6, PMID: 32702296

[2] Brauner JM Mindermann S Sharma M Johnston D Salvatier J Gavenčiak T. Inferring the effectiveness of government interventions against COVID-19. Science. (2021) 371:eabd9338. doi: 10.1126/science.abd9338, PMID: 33323424 PMC7877495

[3] De León EA Shriwise A Tomson G Morton S Lemos DS Menne B. Beyond building back better: imagining a future for human and planetary health. Lancet Planet Health. (2021) 5:e827–39. doi: 10.1016/S2542-5196(21)00262-X, PMID: 34774123 PMC8600369

[4] Tavakkoli M Karim A Fischer FB Monzon Llamas L Raoofi A Zafar S. From public health policy to impact for COVID-19: a multi-country case study in Switzerland, Spain, Iran and Pakistan. Int J Public Health. (2022) 67:171. doi: 10.3389/ijph.2022.1604969, PMID: 36119450 PMC9472296

[5] Osterrieder A Cuman G Pan-Ngum W Cheah PK Cheah P-K Peerawaranun P. Economic and social impacts of COVID-19 and public health measures: results from an anonymous online survey in Thailand, Malaysia, the UK, Italy, and Slovenia. BMJ Open. (2021) 11:e046863. doi: 10.1136/bmjopen-2020-046863, PMID: 34285007 PMC8295020

[6] Riehm KE Badillo Goicoechea E Wang FM Kim E Aldridge LR Lupton-Smith CP. Association of non-Pharmaceutical Interventions to reduce the spread of SARS-CoV-2 with anxiety and depressive symptoms: a multi-National Study of 43 countries. Int J Public Health. (2022) 67:20. doi: 10.3389/ijph.2022.1604430, PMID: 35308051 PMC8927027

[7] Liu Y Morgenstern C Morgenstern C Kelly J Lowe R Jit M. The impact of non-pharmaceutical interventions on SARS-CoV-2 transmission across 130 countries and territories. BMC Med. (2021) 19:40. doi: 10.1186/s12916-020-01872-8, PMID: 33541353 PMC7861967

[8] Centers for Disease Control and Prevention, CDC. Nonpharmaceutical interventions (NPIs). (2022) Available at: https://www.cdc.gov/nonpharmaceutical-interventions/index.html (Accessed March 28, 2023)

[9] Flaxman S Mishra S Gandy A Unwin HJT Mellan TA Coupland H. Estimating the effects of non-pharmaceutical interventions on COVID-19 in Europe. Nature. (2020) 584:257–61. doi: 10.1038/s41586-020-2405-7 32512579

[10] Lison A Banholzer N Sharma M Mindermann S Unwin HJT Mishra S. Effectiveness assessment of non-pharmaceutical interventions: lessons learned from the COVID-19 pandemic. Lancet Public Health. (2023) 8:e311–7. doi: 10.1016/S2468-2667(23)00046-4, PMID: 36965985 PMC10036127

[11] Banholzer N Van Weenen E Lison A Cenedese A Seeliger A Kratzwald B. Estimating the effects of non-pharmaceutical interventions on the number of new infections with COVID-19 during the first epidemic wave. PLoS One. (2021) 16:e0252827. doi: 10.1371/journal.pone.0252827, PMID: 34077448 PMC8171941

[12] Davies NG Kucharski AJ Eggo RM Gimma A Edmunds WJ Jombart T. Effects of non-pharmaceutical interventions on COVID-19 cases, deaths, and demand for hospital services in the UK: a modelling study. Lancet Public Health. (2020) 5:e375–85. doi: 10.1016/S2468-2667(20)30133-X, PMID: 32502389 PMC7266572

[13] Zhong L Diagne M Wang W Gao J. Country distancing increase reveals the effectiveness of travel restrictions in stopping COVID-19 transmission. Communications. Physics. (2021) 4:1–12. doi: 10.1038/s42005-021-00620-5

[14] Cepaluni G Dorsch MT Kovarek D. Mobility and policy responses during the COVID-19 pandemic in 2020. Int J Public Health. (2022) 67:142. doi: 10.3389/ijph.2022.1604663, PMID: 35990190 PMC9389530

[15] Ye L Li WF Shao J Xu Z Ju J Xu H. Fighting omicron epidemic in China: real-world big data from Fangcang shelter hospital during the outbreak in Shanghai 2022. J Inf Secur. (2022) 85:436–80. doi: 10.1016/j.jinf.2022.07.006, PMID: 35835412 PMC9273521

[16] Lewis D. The next worrisome coronavirus variant could come from China-will it get detected? Nature. (2023). doi: 10.1038/d41586-023-00112-2 36693967

[17] Liu J Liu M Liang W. The dynamic COVID-zero strategy in China. China CDC Weekly. (2022) 4:74–5. doi: 10.46234/ccdcw2022.015, PMID: 35186372 PMC8837441

[18] Liang WN Liu M Liu J Wang YD Wu J Liu X. The dynamic COVID-zero strategy on prevention and control of COVID-19 in China. Zhonghua Yi Xue Za Zhi. (2022) 102:239–42. doi: 10.3760/cma.j.cn112137-20211205-02710, PMID: 35073672

[19] Zhou L Yan W Li S Yang H Zhang X Lu W. Cost-effectiveness of interventions for the prevention and control of COVID-19: systematic review of 85 modelling studies. Journal of. Glob Health. (2022) 12:12. doi: 10.7189/jogh.12.05022, PMID: 35712857 PMC9196831

[20] König M Winkler A. The impact of government responses to the COVID-19 pandemic on GDP growth: does strategy matter? PLoS One. (2021) 16:e0259362. doi: 10.1371/journal.pone.0259362, PMID: 34739509 PMC8570518

[21] Milani F. COVID-19 outbreak, social response, and early economic effects: a global VAR analysis of cross-country interdependencies. J Popul Econ. (2021) 34:223–52. doi: 10.1007/s00148-020-00792-4, PMID: 32839640 PMC7437387

[22] Kotoky S.V.P.A., China’s Covid absolutism makes it a no-go zone for airlines., in Bloomberg. (2022), Bloomberg. Available at: https://www.bloomberg.com/news/articles/2022-01-13/china-s-covid-absolutism-is-making-it-a-no-go-zone-for-airlines (Accessed March 28, 2023).

[23] World Tourism Organization Yearbook of Tourism Statistics. (2020) Available at: https://www.statista.com/statistics/234785/international-tourists-arrivals-in-china/ (Accessed March 28, 2023)

[24] Wilson N Baker MG Blakely T Eichner M. Estimating the impact of control measures to prevent outbreaks of COVID-19 associated with air travel into a COVID-19-free country. Sci Rep. (2021) 11:10766. doi: 10.1038/s41598-021-89807-y, PMID: 34031465 PMC8144219

[25] China CDC Weekly report, Tracking the Epidemic. (2021). Available at: https://weekly.chinacdc.cn/news/TrackingtheEpidemic2021.htm (Accessed May 17, 2023).

[26] Li Z Liu F Cui J Peng Z Chang Z Lai S. Comprehensive large-scale nucleic acid–testing strategies support China’s sustained containment of COVID-19. Nat Med. (2021) 27:740–2. doi: 10.1038/s41591-021-01308-7, PMID: 33859409

[27] Grépin KA Ho T-L Liu Z Marion S Piper J Worsnop CZ. Evidence of the effectiveness of travel-related measures during the early phase of the COVID-19 pandemic: a rapid systematic review. BMJ Glob Health. (2021) 6:e004537. doi: 10.1136/bmjgh-2020-004537, PMID: 33722793 PMC7969755

[28] Chinazzi M Davis JT Ajelli M Gioannini C Litvinova M Merler S. The effect of travel restrictions on the spread of the 2019 novel coronavirus (COVID-19) outbreak. Science. (2020) 368:395–400. doi: 10.1126/science.aba9757, PMID: 32144116 PMC7164386

[29] National Center for Immunization and Respiratory Diseases (NCIRD) Interim infection prevention and control recommendations for healthcare personnel during the coronavirus disease 2019 (COVID-19) pandemic. September 23, 2022. Available at: https://www.cdc.gov/coronavirus/2019-ncov/hcp/infection-control-recommendations.html (Accessed March 28, 2023).

[30] Chen Z Peng Y Wu X Pang B Yang F Zheng W. Comorbidities and complications of COVID-19 associated with disease severity, progression, and mortality in China with centralized isolation and hospitalization: a systematic review and meta-analysis. Front Public Health. (2022) 10:923485. doi: 10.3389/fpubh.2022.923485, PMID: 36052001 PMC9424916

[31] Zhu P Tan X. Is compulsory home quarantine less effective than centralized quarantine in controlling the COVID-19 outbreak? Evidence from Hong Kong. Sustain Cities Soc. (2021) 74:103222. doi: 10.1016/j.scs.2021.103222, PMID: 34367885 PMC8327569

[32] Grijalva CG Rolfes MA Zhu Y McLean HQ Hanson KE Belongia EA. Transmission of SARS-COV-2 infections in households—Tennessee and Wisconsin, April–September 2020. Morb Mortal Wkly Rep. (2020) 69:1631. doi: 10.15585/mmwr.mm6944e1, PMID: 33151916 PMC7643897

[33] Chen Y-C Lu P-E Chang C-S Liu T-H. A time-dependent SIR model for COVID-19 with undetectable infected persons. IEEE Trans Netw Sci Eng. (2020) 7:3279–94. doi: 10.1109/TNSE.2020.3024723 PMC8769021 37981959

[34] Calafiore GC Novara C Possieri C. A time-varying SIRD model for the COVID-19 contagion in Italy. Annu Rev Control. (2020) 50:361–72. doi: 10.1016/j.arcontrol.2020.10.005, PMID: 33132739 PMC7587010

[35] Spannaus A Papamarkou T Erwin S Christian JB. Inferring the spread of COVID-19: the role of time-varying reporting rate in epidemiological modelling. Sci Rep. (2022) 12:10761. doi: 10.1038/s41598-022-14979-0, PMID: 35750796 PMC9232503

[36] Zhang Y Zou B Zhang H Zhang J. Empirical research on male preference in China: a result of gender imbalance in the seventh population census. Int J Environ Res Public Health. (2022) 19:6482. doi: 10.3390/ijerph19116482, PMID: 35682066 PMC9180325

[37] United Nation, International Think Tank for Landlocked Developing Countries (LLDCs), Impact of covid-19 and responses in landlocked developing countries (2021). United nation. Available at: https://www.un.org/ohrlls/sites/www.un.org.ohrlls/files/impact_of_covid19_and_responses_in_lldcs.pdf (Accessed March 28, 2023).

[38] Michlmayr D Hansen CH Gubbels SM Valentiner-Branth P Bager P Obel N. Observed protection against SARS-CoV-2 reinfection following a primary infection: a Danish cohort study among unvaccinated using two years of nationwide PCR-test data. Lancet Reg Health. (2022) 20:100452. doi: 10.1016/j.lanepe.2022.100452, PMID: 35791335 PMC9245510

[39] Ding X Huang S Leung A Rabbany R. Incorporating dynamic flight network in SEIR to model mobility between populations. Applied network. Appl Netw Sci. (2021) 6:42. doi: 10.1007/s41109-021-00378-3, PMID: 34150986 PMC8205202

[40] Balsa C Lopes I Guarda T Rufino J. Computational simulation of the COVID-19 epidemic with the SEIR stochastic model. Comput Math Organ Theory. (2021) 20:1–19. doi: 10.1007/s10588-021-09327-y, PMID: 33814968 PMC8007662

[41] Tang B Wang X Li Q Bragazzi NL Tang S Xiao Y. Estimation of the transmission risk of the 2019-nCoV and its implication for public health interventions. J Clin Med. (2020) 9:462. doi: 10.3390/jcm9020462, PMID: 32046137 PMC7074281

[42] Qiu Z Sun Y He X Wei J Zhou R Bai J. Application of genetic algorithm combined with improved SEIR model in predicting the epidemic trend of COVID-19, China. Sci Rep. (2022) 12:8910. doi: 10.1038/s41598-022-12958-z, PMID: 35618751 PMC9133826

[43] Brown RA. A simple model for control of COVID-19 infections on an urban campus. Proc Natl Acad Sci U S A. (2021) 118:e2105292118. doi: 10.1073/pnas.2105292118, PMID: 34475214 PMC8433581

[44] Kwuimy CAK Nazari F Jiao X Rohani P Nataraj C. Nonlinear dynamic analysis of an epidemiological model for COVID-19 including public behavior and government action. Nonlinear Dynamics. (2020) 101:1545–59. doi: 10.1007/s11071-020-05815-z, PMID: 32836814 PMC7363774

[45] McGee RS Homburger JR Williams HE Bergstrom CT Zhou AY. Model-driven mitigation measures for reopening schools during the COVID-19 pandemic. Proc Natl Acad Sci. (2021) 118:e2108909118. doi: 10.1073/pnas.2108909118, PMID: 34518375 PMC8488607

[46] Kharazmi E Cai M Zheng X Zhang Z Lin G Karniadakis GE. Identifiability and predictability of integer- and fractional-order epidemiological models using physics-informed neural networks. Nat Comput Sci. (2021) 1:744–53. doi: 10.1038/s43588-021-00158-0 38217142

[47] Kucharski AJ Russell TW Diamond C Liu Y Edmunds J Funk S. Early dynamics of transmission and control of COVID-19: a mathematical modelling study. Lancet Infect Dis. (2020) 20:553–8. doi: 10.1016/S1473-3099(20)30144-4, PMID: 32171059 PMC7158569

[48] Frazier PI Cashore JM Duan N Henderson SG Janmohamed A Liu B. Modeling for COVID-19 college reopening decisions: Cornell, a case study. Proc Natl Acad Sci. (2022) 119:e2112532119. doi: 10.1073/pnas.2112532119, PMID: 34969678 PMC8764692

[49] Disease Control Priorities. Disease control priorities: improving health and reducing poverty. In: Jamison DT Gelband H Horton S Jha P Laxminarayan R Mock CN, editors. Disease Control Priorities, vol. 9. 3rd ed: Washington, DC: World Bank Group (2017) 30212058

[50] Zhang X Yan B. Climate change and city size: the role of temperature difference in the spatial distribution of China’s population. Environ Sci Pollut Res. (2022) 29:82232–42. doi: 10.1007/s11356-022-21561-8, PMID: 35748990

[51] Chen C Feng Y Chen Z Xia Y Zhao X Wang J. SARS-CoV-2 cold-chain transmission: characteristics, risks, and strategies. J Med Virol. (2022) 94:3540–7. doi: 10.1002/jmv.27750, PMID: 35355277 PMC9088485

[52] Li Y-Y Liu H-X Xia W Wong GWK Xu S-Q. Cold chain logistics: a possible mode of SARS-CoV-2 transmission? BMJ. (2021) 375:e066129. doi: 10.1136/bmj-2021-066129, PMID: 34853016 PMC9394745

[53] Zhao H Lu X Lun W Li T Rao B Wang D. Transmission dynamics of SARS-CoV-2 in a mid-size city of China. BMC Infect Dis. (2021) 21:793–9. doi: 10.1186/s12879-021-06522-9, PMID: 34376168 PMC8353423

[54] Choi S Jung E. Optimal tuberculosis prevention and control strategy from a mathematical model based on real data. Bull Math Biol. (2014) 76:1566–89. doi: 10.1007/s11538-014-9962-6, PMID: 24849770

[55] Hao X Cheng S Wu D Wu T Lin X Wang C. Reconstruction of the full transmission dynamics of COVID-19 in Wuhan. Nature. (2020) 584:420–4. doi: 10.1038/s41586-020-2554-8, PMID: 32674112

[56] National Health Commission of the People’s Republic of China Protocol for prevention and control of COVID-19 (edition 9), Center for Disease Control and Prevention. (2022) National Health Commission: The State Council the People’s Republic of China. Available at: https://weekly.chinacdc.cn/ (Accessed March 28, 2023). 10.46234/ccdcw2020.082 PMC8392946 34594648

[57] Ferguson NM Laydon D Nedjati-Gilani G Imai N Ainslie K Baguelin M. Impact of non-pharmaceutical interventions (NPIs) to reduce COVID-19 mortality and healthcare demand. Imperial college COVID-19 response team. Imper Coll COVID-19 Resp Team. (2020) 20:77482. doi: 10.25561/77482

[58] Errett NA Sauer LM Rutkow L. An integrative review of the limited evidence on international travel bans as an emerging infectious disease disaster control measure. Am J Disaster Med. (2019) 18:7–14. doi: 10.5055/jem.2020.0446, PMID: 32421851

[59] Le T-M Raynal L Talbot O Hambridge H Drovandi C Mira A. Framework for assessing and easing global COVID-19 travel restrictions. Sci Rep. (2022) 12:6985. doi: 10.1038/s41598-022-10678-y, PMID: 35484268 PMC9049014

[60] Kang H Min K-D Jeon S Lee J-Y Cho S-I. A measure to estimate the risk of imported COVID-19 cases and its application for evaluating travel-related control measures. Sci Rep. (2022) 12:9497. doi: 10.1038/s41598-022-13775-0, PMID: 35681085 PMC9178220

[61] Veneti L Bøås H Kristoffersen AB Stålcrantz J Bragstad K Hungnes O. Reduced risk of hospitalisation among reported COVID-19 cases infected with the SARS-CoV-2 omicron BA. 1 variant compared with the Delta variant, Norway, December 2021 to January 2022. Eur Secur. (2022) 27:2200077. doi: 10.2807/1560-7917.ES.2022.27.4.2200077 PMC8796289 35086614

[62] Endo A Abbott S Kucharski AJ Funk S. Estimating the overdispersion in COVID-19 transmission using outbreak sizes outside China. Wellcome Open Res. (2020) 5:67. doi: 10.12688/wellcomeopenres.15842.3, PMID: 32685698 PMC7338915

[63] Lloyd-Smith JO Schreiber SJ Kopp PE Getz WM. Superspreading and the effect of individual variation on disease emergence. Nature. (2005) 438:355–9. doi: 10.1038/nature04153, PMID: 16292310 PMC7094981

